# Immunomodulatory potential of four candidate probiotic *Lactobacillus* strains from plant and animal origin using comparative genomic analysis

**DOI:** 10.1099/acmi.0.000299

**Published:** 2021-12-17

**Authors:** Paul Benedic U. Salvador, Leslie Michelle M. Dalmacio, Sang Hoon Kim, Dae-Kyung Kang, Marilen P. Balolong

**Affiliations:** ^1^​ Department of Biochemistry and Molecular Biology, College of Medicine, University of the Philippines Manila, Ermita, Manila 1000, Philippines; ^2^​ Department of Animal Resources Science, College of Biotechnology and Bioengineering, Dankook University, Republic of Korea; ^3^​ Department of Biology, College of Arts and Sciences, University of the Philippines Manila, Ermita, Manila 1000, Philippines

**Keywords:** probiotics, *Lactobacillus*, comparative genomics, immunomodulation

## Abstract

Probiotic strains from different origins have shown promise in recent decades for their health benefits, for example in promoting and regulating the immune system. The immunomodulatory potential of four *Lactobacillus* strains from animal and plant origins was evaluated in this paper based on their genomic information. Comparative genomic analysis was performed through genome alignment, average nucleotide identity (ANI) analysis and gene mining for putative immunomodulatory genes. The genomes of the four *Lactobacillus* strains show relative similarities in multiple regions, as observed in the genome alignment. However, ANI analysis showed that *L. mucosae* LM1 and *L. fermentum* SK152 are the most similar when considering their nucleotide sequences alone. Gene mining of putative immunomodulatory genes studied from *L. plantarum* WCFS1 yielded multiple results in the four potential probiotic strains, with *L. plantarum* SK151 showing the largest number of genes at around 74 hits, followed by *L. johnsonii* PF01 at 41 genes when adjusted for matches with at least 30 % identity. Looking at the immunomodulatory genes in each strain, *L. plantarum* SK151 and *L. johnsonii* PF01 may have wider activity, covering both immune activation and immune suppression, as compared to *L. mucosae* LM1 and *L. fermentum* SK152, which could be more effective in activating immune cells and the pro-inflammatory cascade rather than suppressing it. The similarities and differences between the four *Lactobacillus* species showed that there is no definitive trend based on the origin of isolation alone. Moreover, higher percentage identities between genomes do not directly correlate with higher similarities in potential activity, such as in immunomodulation. The immunomodulatory function of each of the four *Lactobacillus* strains should be observed and verified experimentally in the future, since some the activity of some genes may be strain-specific, which would not be identified through comparative genomics alone.

## Impact Statement

Probiotics are widely available and starting to be recognized as a functional food because of their potential clinical benefits. Multiple studies have demonstrated the potential health benefits of probiotics, as these can improve gut absorption and may improve the immune system. However, there is still a gap in understanding how these probiotic strains exert health benefits to their hosts. Looking closely at the immunomodulatory potential of various *Lactobacillus* species may aid in the clinical use and effectiveness of probiotics for diseases involving dysregulation of the immune system. This study may serve as a basis for determining the mechanism of *Lactobacillus* strains for immunomodulation. The insights and results from comparative genomic studies may also guide future studies in their approach to verifying the potential of these strains for immunomodulation and other clinical health benefits.

## Data Summary

All whole-genome sequence data for the four *Lactobacillus* species were obtained using the National Center for Biotechnology Information’s GenBank database with the following accession numbers: CP011013.1, CP030105.1, CP016803.1 and CP024781.1. The authors confirm that all supporting data, code and protocols have been provided within the article or through supplementary data files.

## Introduction

The role of functional food has been an emerging topic for research in the past decade. Functional food is differentiated from food, as the latter is taken for the sole purpose of nutrition, while the former may confer physical and mental health benefits on humans [1]. Probiotics are included in these functional foods and are composed of live micro-organisms that may be ingested or taken by humans, leading to a health benefit [1, 2]. Various strains of bacteria have gained recognition as probiotics, such as different *Lactobacillus* and *Bifidobacterium* species [1, 3, 4]. Studies were performed in order to determine how these microbes can benefit their hosts. Various studies showed that probiotics may have various health benefits, such as better gastrointestinal function and absorption, decreased colonization of pathogenic bacteria, and improved immune response [3–6]. Probiotics have even shown promising results as adjunct intervention for diseases such as antibiotic-associated diarrhoea, infectious diarrhoea and inflammatory bowel disease [4, 7, 8]. In terms of their immunomodulatory properties, probiotics have also also showed promise, as these have been observed to induce IgA secretion in the gastrointestinal mucosa, modulate cytokine profiles and regulate T cell polarity between Th1 and Th2 response [4, 9–15]. However, despite multiple studies on probiotics as functional food, their clinical use is still limited and their exact mechanisms of action have yet to be demonstrated.

Functional genomics has been used for probiotics to determine putative genes that could explain the capacity of these microbes to have antimicrobial activity or to promote gastrointestinal health [16]. Comparative genomics were also applied to look at different probiotic species, since some have higher virulence factors, while others are associated with more clinical benefit than others [17]. Even within the same species, comparative genomics may be applied, such as in the case of different *Lactobacillus plantarum* strains. Studies have shown that *L. plantarum* WCFS1, ZJ316 and ST-III could have immunomodulatory properties due to the presence of various capsular polysaccharide biosynthesis genes, which may be vital for colonization and host–bacteria interaction with immune cells [18]. Plantaricin biosynthesis genes also showed potential for immunomodulatory properties through cytokine induction, and these were abundant in *L. plantarum* WCFS1, NC8, ZJ316 and ST-III [18, 19]. Differences in isolation sources of probiotic strains may also be vital in genomic analysis, since studies have shown that strains isolated from relatively friendly environments such as milk, cheese, food and the healthy human gut have fewer virulence factors and are associated with less risk compared to probiotics isolated from patient specimens, soil and silage [17]. It is then worth exploring and studying different *Lactobacillus* species that have been isolated from animal and plant origins, particularly in their potential mechanisms and application for immunomodulation.

In this preliminary study, comparative genomic analysis was performed for four *Lactobacillus* strains, namely *L. mucosae* LM1 (CP011013.1), *L. plantarum* SK 151 (CP030105.1), *L. fermentum* SK 152 (CP016803.1) and *L. johnsonii* PF01 (CP024781.1), which were isolated from animal and plant origins. These strains were chosen because these were previously evaluated by the same research group, showing great probiotic potential [20–32]. This study aims to further expand current knowledge of these strains and assess their probiotic potential, particularly for immunomodulation. The genome data for all strains were obtained from a public database and were compared through genome alignment and average nucleotide identity, as well as through gene mining, in order to determine the similarities and differences between each genome, particularly with respect to its genes with potential for immunomodulatory activity.

## Methodology

### Comparative genome analysis

All the genome data for the *Lactobacillus* strains were obtained from the The National Center for Biotechnology Information’s (the NCBI’s) GenBank database using the accession numbers CP011013.1, CP030105.1, CP016803.1 and CP024781.1, and were processed using Mauve software v. 2.3.1 through progressiveMauve [33]. Genome alignment was performed by determining locally colinear blocks on the whole genomes. Iterative refinement was also included for increased accuracy between the alignment and scoring. Using the software developed by Lee *et al*. in 2015, ANI analysis was also performed [34]. The Orthologous Average Nucleotide Identity Tool (OAT), which uses OrthoANI, was used to analyse the genomes of four *Lactobacillus* species and calculate the similarities between each based primarily on their sequences [34].

Using the annotated genomes of the four *Lactobacillus* strains, genes with putative immunomodulatory activities were mined using command line blast 2.2.30+ with the e-value set to <0.00001 for similarity and accuracy and max target sequences set to 1 [35]. Since there are no databases available for immunomodulatory genes in probiotic strains, all the genes that were mined in the genomes were based on the previous literature. These genes were studied to have putative immunomodulatory function, which may directly or indirectly influence the immune response of the host. The presence of homologues of identified immunomodulatory genes and products was determined visually through genome alignment with progressiveMauve in Mauve software v. 2.3.1 [33]. Annotated genomes were aligned and searched with the mined immunomodulatory genes, particularly bacteriocin and plantaricin protein components.

## Results and Discussion

### Genome alignment

**Fig. 1.**
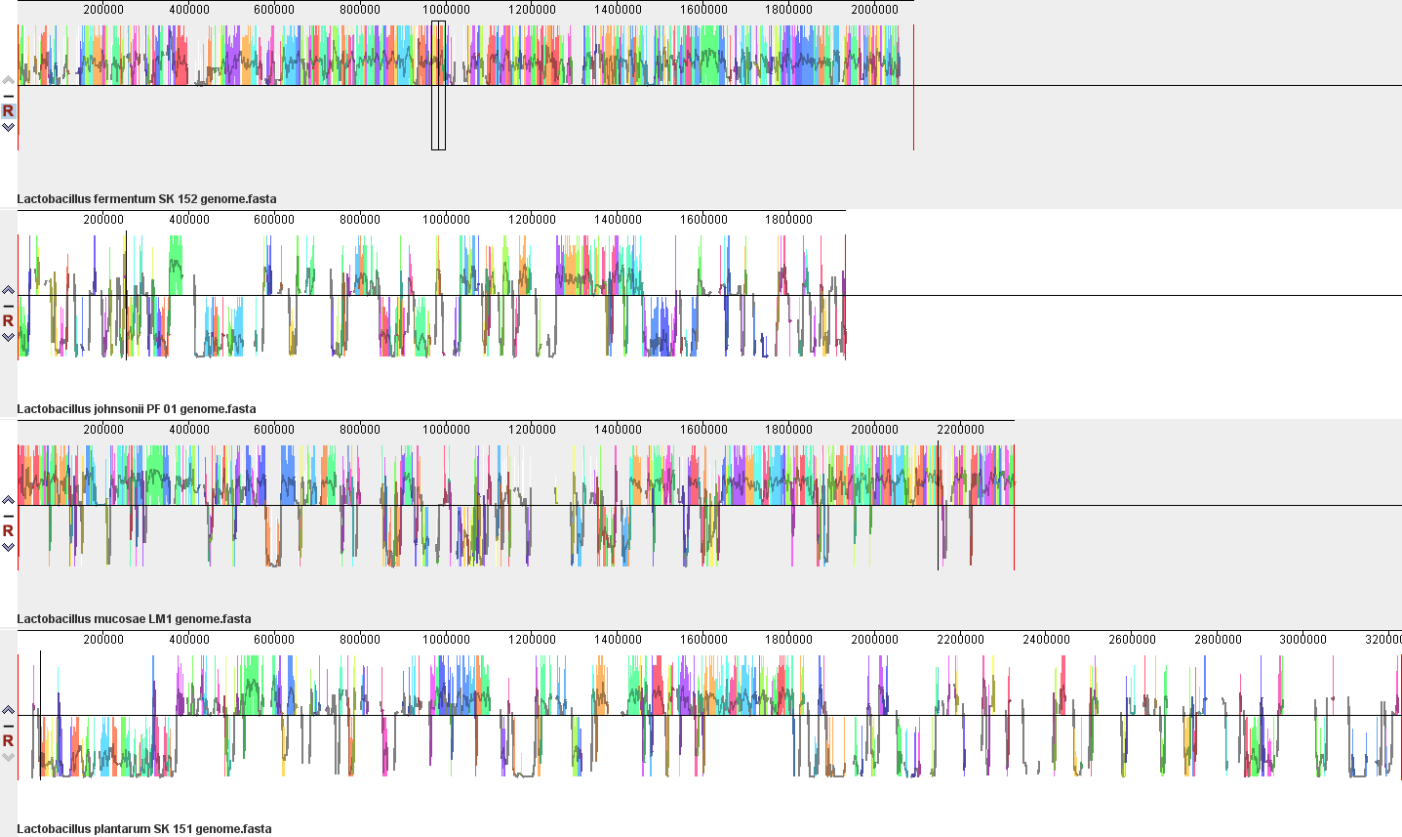
Genome alignment of the four *Lactobacillus* strains using Mauve software v. 2.3.1. Locally colinear blocks are indicated by the highlighted regions of the same respective colours among the genomes. Mean similarity plots and ranges are indicated by the darker coloured lines inside each locally colinear block.

Comparison between the four genomes of *Lactobacillus* strains showed relatively high similarity in the different genes present in the genome. Regions highlighted with the same colours indicate locally colinear blocks among the genomes that are regions with homologous backbone sequences (Fig. 1). The presence of these indicates that there are multiple conserved regions in the genomes of the four strains. However, looking at the mean similarity plots as indicated by the dark lines in each locally colinear block, the sequences of the genomes have a much variability (Fig. 1). These variations between the genomes may indicate differences in the degree of function or activity of genes between species despite the presence of conserved regions.

### Average nucleotide identity (ANI) analysis

To better characterize the similarities and differences between the sequences of the genomes, ANI analysis was performed using OAT or OrthoANI [34]. The percentages of similarities can be observed with the proposed cut-off between species set at 95–96 % (Table 1). Since the four *Lactobacillus* strains are from different species, a significantly high degree of similarity is not expected. However, among all the different species, the closest ones are *L. mucosae* LM1 and *L. fermentum* SK152, with 70.36 % similarity. This result is interesting, since *L. mucosae* LM1 was isolated in the small intestine of a swine, which is the same as for *L. johnsonii* PF01, which was also isolated from a pig [31, 36]. The similarities of *L. plantarum* SK151 and *L. fermentum* SK152 are also higher for *L. mucosae* LM1, despite these two strains being isolated from kimchi, a traditional Korean fermented food [32, 37]. Despite similarities in their origins, *L. plantarum* SK151 and *L. fermentum* SK152 only have 65.93 % similarity. Hence, similarities in the source or origin of potential probiotic species alone may not show direct correlation in their sequences. Moreover, *Lactobacillus* strains with higher ANI may also come from different sources.

**Table 1.** Similarities between nucleotide sequences of four *Lactobacillus* strains in the whole genome (shown in percentages) using ANI analysis

	LM 1	SK 151	SK 152	PF 01
* L. mucosae * LM 1	–	66.074 %	70.36 %	65.51 %
* L. plantarum * SK 151	66.074 %	–	65.93 %	65.36 %
* L. fermentum * SK 152	70.36 %	65.93 %	–	64.36 %
* L. johnsonii * PF 01	65.51 %	65.36 %	64.36 %	–

Recently, the genus *Lactobacillus* has been evaluated and a taxonomic reclassification was proposed [38]. Under this new classification, *L. mucosae* and *L. fermentum* were both reclassified under the genus *Limosilactobacillus *, while *L. plantarum* was reclassified under *Lactiplantibacillus* and *L. johnsonii* remained unchanged under the amended genus *Lactobacillus* [38]. This new taxonomic classification may explain why *L. mucosae* LM1 and *L. fermentum* SK152 have the highest similarities in ANI analysis, since both of these species will be reclassified under *Limosilactobacillus *.

### Gene mining for immunomodulatory genes

A literature review was performed in order to determine putative immunomodulatory genes in *Lactobacillus* strains. These genes were summarized based on the results obtained from reference genome organisms, mainly *L. plantarum* WCFS1 (Table 2). Promising results were obtained, as these genes were able to either promote immune activation through activation of macrophages and dendritic cells, polarization towards Th1 response of T-lymphocytes, or suppression of immune activation via induction of IL-10 [19, 39, 40]. Hence, these genes were mined in the genomes of the four *Lactobacillus* species in order to determine the presence of these putative immunomodulatory genes. Currently, there is no database for immunomodulatory genes. Most studies on immunomodulation also focus on the phenotypic effect of probiotic strains and do not directly identify putative genes that can activate or repress the immune system. Therefore, the reference immunomodulatory genes used in this research have been heavily based on studies using *L. plantarum* WCFS1.

**Table 2.** Immunomodulatory genes based on *L. plantarum* WCFS1 studies

Genes	Function	Immunomodulatory activity	References
*lp_2991*	Transcription regulator	Increase in IL-10 and TNF- α	[19]
*plnE*	Bacteriocin-like peptide E	Increase in IL-10	
*plnF*	Bacteriocin-like peptide F		
*plnI*	Bacteriocin immunity protein		
*plnG*	Bacteriocin ABC transporter, ATP-binding and permease protein	Increase in IL-10	
*plnH*	Bacteriocin ABC transporter, accessory factor	Increase in IL-10	
*plnS*	Plantaricin biosynthesis protein		
*plnTUVW*	Hypothetical membrane proteins		
*bsh1*	Bile salt hydrolase	Increase in TNF- α	
*cps* gene cluster (*cps1A to cps1I*) (*cps 2A to cps 2K*) (*cps3A to cps3J*) (*cps4A to cps4J*)	Synthesis of capsule polysaccharides and exopolysaccharides (biosynthesis proteins, glycosyltransferases, oligosaccharide transporters, acyl/acetyltransferases, polymerases, epimerases, phosphatase and polysaccharide repeat unit transporter)	Increase in IL-6, IL-1β, and TNF-α Increase in phagocytosis and NO production	[39, 40]

Considering all the genes in the genomes of the four strains, the highest number of immunomodulatory genes can be observed in *L. plantarum* SK151, reaching as high as 155 genes (Table 3). The highest percentage identity was also observed in this genome, with 100 % identity in 13 genes. This is expected, since the reference genome used in the literature to study the putative immunomodulatory genes is *L. plantarum* WCFS1, which is the same species as one of the four potential probiotic strains being studied. Considering the other species, *L. johnsonii* PF01 showed the second greatest number of putative immunomodulatory genes, with 91 genes. However, since some of the genes have very low percentage identity, the total number of genes were adjusted by only considering those that have at least 30 % identity with the reference immunomodulatory gene sequences. This adjusted value returned 74 genes and 41 genes for *L. plantarum* SK151 and *L. johnsonii* PF01, respectively (Table 3). Both *L. fermentum* SK152 and *L. mucosae* LM01 are almost similar in the number of immunomodulatory genes, with an adjusted number of 29 and 30, respectively.

**Table 3.** Total and adjusted number of putative immunomodulatory genes found in the four *Lactobacillus* strains

	Total	Adjusted
* L. mucosae* LM1	65 (22.27 %–61.26 %)	30
* L. plantarum* SK151	155 (20.71 %–100 %)	74
* L. fermentum* SK152	68 (21.25 %–75.45 %)	29
* L. johnsonii* PF01	91 (20.43 %–62.34 %)	41

All of the matches genes that have e-values <0.00001 compared to the FASTA sequences of the immunomodulatory genes from *L. plantarum* WCFS1 strain. Values inside the parenthesis indicate the range of the percentge identity of the genes mined in the respective genomes.

Looking at the immunomodulatory genes with the highest percentage identities in each strain, some insights may be obtained (Table 4). The complete list of mined putative immunomodulatory genes may be found in the Table S1 (available in the online version of this article). Considering the case of *L. plantarum* SK151, the genes with the highest percentage identities include bacteriocin-related proteins and peptides such as bacteriocin ABC transporters, plantaricin biosynthesis protein and membrane proteins, as well as some of the proteins included in the *cps* gene cluster for capsule or exopolysaccharides. The presence of different bacteriocin genes or proteins may vary, depending on the strain, as these changes also pertain to differences in the structure of their capsule, such as variation in the branching and composition of the sugar backbone present [41]. All the different bacteriocin genes are not necessary to synthesize a capsular polysaccharide and therefore variation among different potential probiotic strains may be observed. Bacteriocin and plantaricin components were studied to have potential for immunomodulation due to its ability to trigger TLR response in dendritic cells and T cells, inducing production of cytokines like IL-10 and TNF- α [19]. Aside from this, bacteriocin and plantaricin may also help regulate immune activation within the gut, since these are antimicrobial peptides, which can help the survival of the probiotic while limiting the growth of other bacterial strains [18]. Some *in vitro* studies even show that these bacteriocins may be absorbed in vascular epithelial cells, highlighting their potential for systemic immunomodulatory activity [42]. Capsules or exopolysaccharides have also shown potential for immunomodulation, since these can induce host–bacteria interactions by activating NF- κ B to promote macrophage activity and by triggering TLR2 and inducing the production of pro-inflammatory cytokines such as IL-6 and TNF- α [18, 39]. However, research has also shown that capsule polysaccharides may also have an anti-inflammatory mechanism, such as in the cases of *L. casei* Shirota strain, *L. paraplantarum* and *L. rhamnosus *, where these genes induce IL-10 and other anti-inflammatory cytokines while also reducing pro-inflammatory cytokines and response of macrophages, dendritic cells and T cells [39, 40]. Given the function of these genes, it can be seen that *L. plantarum* SK151 has a high capacity to mimic the ability of *L. plantarum* WCFS1, which can exert its activity in activating as well as dampening the immune response through the release of pro-inflammatory or anti-inflammatory cytokines [40].

**Table 4.** List of putative immunomodulatory genes in the four *Lactobacillus* species

Strain	Sequence ID	Gene	Immunomodulatory Activity	Percent identity
* L. mucosae * LM1	F9UMX8_LACPL	Glycosyltransferase	Increase in IL-6, IL-1β and TNF-α Increase in phagocytosis and NO production [39, 40]	61.26 %
	B9V401_LACPN	Polysaccharide biosynthesis protein, regulator		50.45 %
	F9UMX7_LACPL	Oligosaccharide transporter (flippase)		45.54 %
	F9UMZ9_LACPL	Priming glycosyltransferase, polyprenyl glycosylphosphotransferase		46 %
	F9UMZ7_LACPL	Tyrosine protein phosphatase		45.49 %
* L. plantarum* SK151	F9UU06_LACPL	Bacteriocin immunity protein	Increase in IL-10 [19]	100 %
	F9UU07_LACPL	Bacteriocin peptide		100 %
	F9UU08_LACPL	Bacteriocin peptide		100 %
	F9UU09_LACPL	Bacteriocin ABC transporter, ATP-binding and permease protein		100 %
	F9UU10_LACPL	Bacteriocin ABC transporter, accessory factor		100 %
* L. fermentum* SK152	F9UMZ9_LACPL	Priming glycosyltransferase, polyprenyl glycosylphosphotransferase	Increase in IL-6, IL-1β and TNF-α Increase in phagocytosis and NO production [39, 40]	75.45 %
	F9UMX8_LACPL	Glycosyltransferase		57.6 %
	F9UQ52_LACPL	UDP N-acetyl glucosamine 4-epimerase		57.52 %
	F9UMZ6_LACPL	Polysaccharide biosynthesis protein, regulator		51.92 %
	F9UMX7_LACPL	Oligosaccharide transporter (flippase)		45.44 %
* L. johnsonii* PF01	F9UU09_LACPL	Bacteriocin ABC transporter, ATP-binding and permease protein	Increase in IL-10 [19]	62.34 %
	B9V401_LACPN	Bile salt hydrolase	Increase in TNF- α [19]	53.16 %
	F9UMZ7_LACPL	Tyrosine protein phosphatase	Increase in IL-6, IL-1β and TNF-α Increase in phagocytosis and NO production [39, 40]	47.06 %
	F9UMZ6_LACPL	Polysaccharide biosynthesis protein, regulator		45.58 %
	F9UMZ9_LACPL	Priming glycosyltransferase, polyprenyl glycosylphosphotransferase		42.34 %

These are some of genes mined from the four strains that showed the highest percentage identity. Only the top five genes were listed for each strain.

**Fig. 2.**
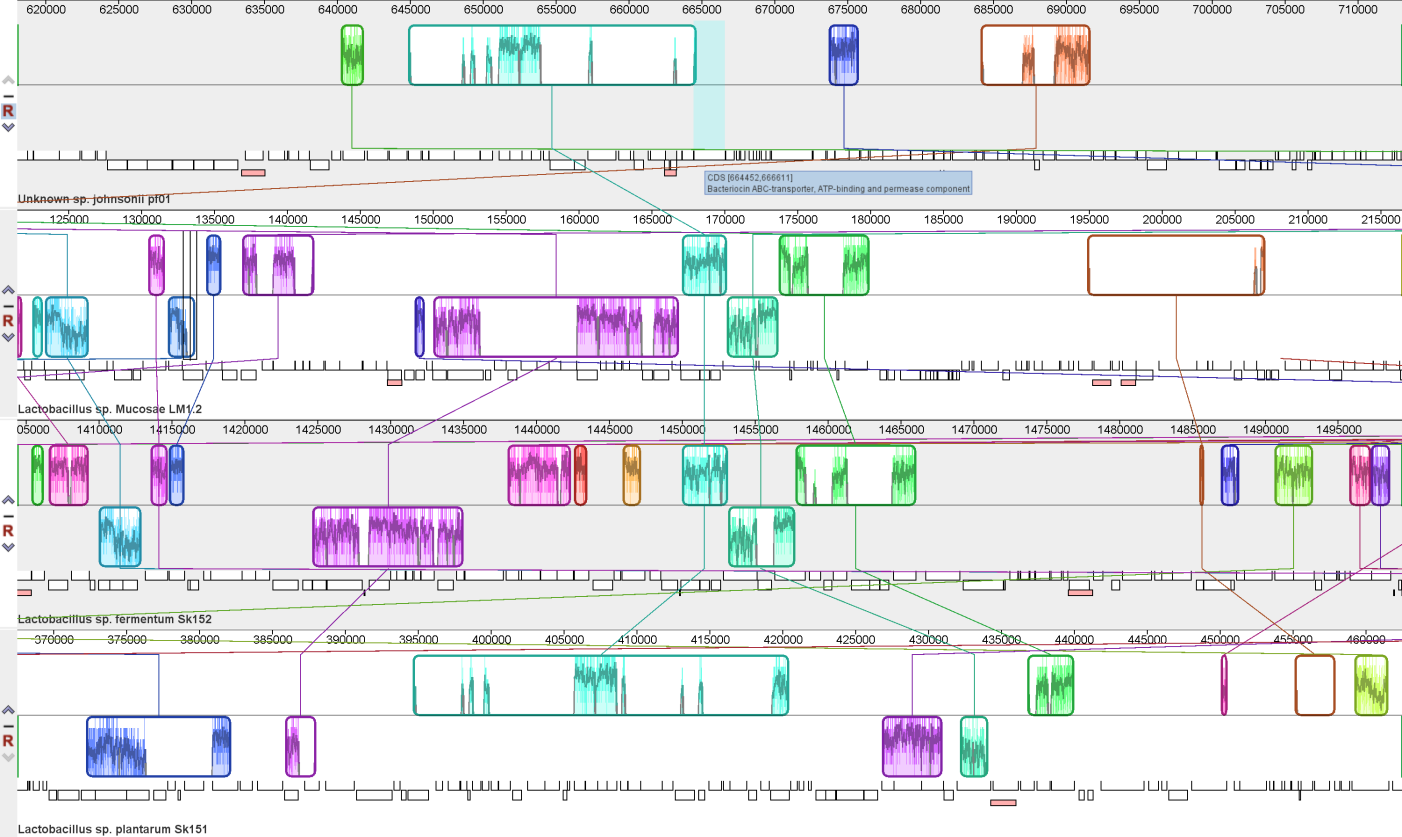
Genome alignment showing the location of the bacteriocin ABC transporter protein component in *Lactobacillus johnsonii* PF01. The gene of the bacteriocin transporter is adjacent to identified locally colinear blocks (indicated by the cyan box) among the genomes based on genome alignment. The locally colinear blocks were observed in all the other *Lactobacillus* species, but the specific bacteriocin transporter gene was only identified in *L. johnsonii* PF01.

**Fig. 3.**
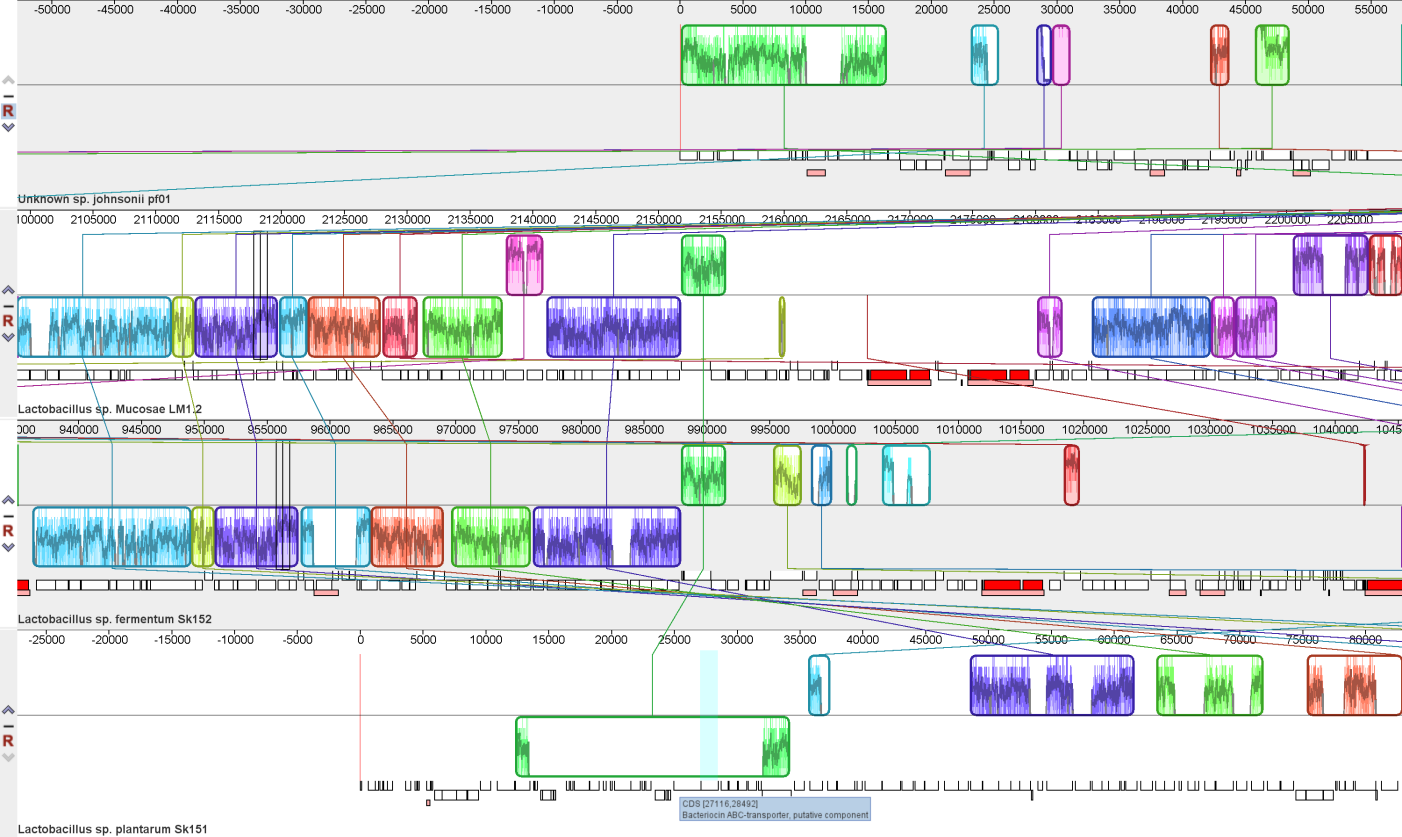
Genome alignment showing the location of the bacteriocin ABC transporter protein component in *Lactobacillus plantarum* SK151. The gene of the bacteriocin transporter is located inside the identified locally colinear blocks (indicated by the green box) among the genomes based on genome alignment. The locally colinear blocks were observed in all the other *Lactobacillus* species, but the specific bacteriocin transporter gene was only seen in the genome of *L. plantarum* SK151.

Immunomodulatory genes with high percentage identities mined in *L. johnsonii* PF01 include both bacteriocin and capsule polysaccharide components, similar to *L. plantarum* SK151. These indicate that *L. johnsonii* PF01 may also have the potential for both immune activation and suppression. However, for *L. mucosae* LM1 and *L. fermentum* SK152, no bacteriocin or plantaricin genes with high fidelity were mined in the genome. Using the annotated genome alignment, the absence of homologues of bacteriocin or plantaricin components in *L. mucosae* LM1 and *L. fermentum* SK152 was confirmed, since bacteriocin gene products in *L. johnsonii* PF01 and *L. plantarum* SK151 do not show in locally colinear blocks in the other two strains (Figs 2 and 3). Locally colinear blocks are segments in the genome that are free of rearrangements of homologous sequences [33]. Therefore, investigating these blocks for similarities or differences in the genes can show how similar sequences between genomes may still lead to differences in genes that are necessary for probiotic activity, such as immunomodulation. In *L. johnsonii* PF01, the bacteriocin gene is adjacent to a locally colinear block (Fig. 2). In the case of *L. plantarum* SK151, the bacteriocin gene components, along with other plantaricin biosynthesis components (not labelled), were located inside a block that was locally colinear with those of *L. mucosae* LM1 and *L. fermentum* SK152 (Fig. 3). Despite this, both *L. mucosae* LM1 and *L. fermentum* SK152 only have genes for Na^+^/H^+^ antiporter and DNA helicase in the locally colinear block with the complete absence of all bacteriocin and plantaricin-related genes that was observed in *L. plantarum* SK151. Therefore, the immunomodulatory activity *L. mucosae* LM1 and *L. fermentum* SK151 may most likely rely on other genes and not on bacteriocin- or plantaricin-related components.

Looking at the genes identified in *L. mucosae* LM1 and *L. fermentum* SK152, high percentage identities in immunomodulatory genes that are related more on immune activation were identified (Table 4). However, the immunomodulatory activity of capsule polysaccharides may be strain-specific, as these are pro-inflammatory for *L. plantarum *, but may be anti-inflammatory, such as in *L. rhamnosus* and *L. paraplantarum* [43]. Therefore, the immunomodulatory potential of *L. mucosae* LM1 and *L. fermentum* SK152 may both be pro-inflammatory or anti-inflammatory, depending on their interaction with host immune cells. Nevertheless, considering that these potential probiotic strains do not have bacteriocin- or plantaricin-related genes, both *L. mucosae* LM1 and *L. fermentum* SK152 may be more limited in their immunomodulatory potential compared to *L. johnsonii* PF01 and *L. plantarum* SK151. Considering the sources of these potential probiotic strains, there might be no direct correlation in their activity for immunomodulation. The reference strain *L. plantarum* WCFS1 was isolated from human saliva, while *L. plantarum* SK151 was isolated from a fermented food [32, 40]. Despite clear differences in their origin, putative immunomodulatory genes in *L. plantarum* WCFS1 were still found in *L. plantarum* SK151. Furthermore, few but similar immunomodulatory genes were found in both *L. mucosae* LM1 and *L. fermentum* SK152. Even though *L. mucosae* LM1 and *L. fermentum* SK152 have different origins, the small intestine of a swine and fermented food, respectively, immunomodulatory genes obtained in these two were comparable, as predominance in capsule polysaccharides was observed rather than on bacteriocin and plantaricin genes.

## Conclusion and future perspectives

Probiotic strains may have a multitude of functions that may even encompass complex processes, such as immunomodulation. In this study, it was shown that the four *Lactobacillus* species, despite differences in their genome and nucleotide sequences, may exhibit immunomodulatory activities, given the presence of putative genes necessary for this function. In terms of ANI analysis, *L. mucosae* LM1 was determined to be more similar to *L. fermentum* SK152. Despite *L. mucosae* LM1 and *L. johnsonii* PF01 being isolated from pigs, these two still have lower similarities in their genome sequences. The same observation was noted for *L. plantarum* SK151 and *L. fermentum* SK152, where their similarity is only at 65.93 %, even though both were isolated from a fermented food. Mining of putative immunomodulatory genes showed that *L. plantarum* SK151 and *L. johnsonii* PF01 have the highest potential to have immunomodulatory activity. These two potential probiotic strains also appeared to likely have a wider immunomodulatory activity, covering both immune activation and suppression through the action of bacteriocin- or plantaricin-related genes, as well as capsule exopolysaccharides, compared to *L. mucosae* LM1 and *L. fermentum* SK152, which may be more suited for pro-inflammatory response. Immunomodulatory potential was also observed for both animal and plant-origin probiotics, with no definite trend or predominance of the other, indicating that the source may not be reflective of its capacity to induce immune response. However, further studies should be performed, as the immunomodulatory activity of probiotics may be strain-specific and other putative immunomodulatory genes may not have been covered adequately. Moreover, other bioinformatic tools for comparative genomic analysis may also be used to increase the scope of analysis. Higher throughput methods for scanning genomes may be applied in future research to evaluate the probiotic potential of these *Lactobacillus* species extensively. Nevertheless, the current tools used for this study can adequately achieve objectives that primarily focus on identifying genome similarities and the presence of putative immunomodulatory genes in the selected strains. Overall, comparative genomics is a great avenue in the analysis and prediction of potential genes and functions in the genomes of various probiotic strains that could be used as a guide in understanding how probiotic strains can indeed exert immunomodulatory activities or other functions in hosts.

## Supplementary Data

Supplementary material 1
