# Supplementary material for: Immunomodulatory potential of four candidate probiotic Lactobacillus strains from plant and animal origin using comparative genomic analysis
Source: Access Microbiol. 2021 Dec 17;3(12):000299. doi: 10.1099/acmi.0.000299 (PMC8749136; doi:10.1099/acmi.0.000299)
Supplement: Supplementary material 1 [file acmi-3-0299-s001.pdf]

Supplementary Table. Complete list of putative immunomodulatory genes mined in the genomes of the four candidate probiotic *Lactobacillus* strains.

Strain	Sequence ID	Gene	Percent Identity (%)
L. mucosae LM1	F9UMX8_LACPL	Glycosyltransferase	61.26
	B9V401_LACPN	Bile salt hydrolase	57.99
	F9UMZ6_LACPL	Polysaccharide biosynthesis protein,regulator	50.45
	F9UMX7_LACPL	Oligosaccharide transporter (Flippase)	46.54
	F9UMZ9_LACPL	Priming glycosyltransferase, polyprenyl glycosylphosphotransferase	46
	F9UMZ7_LACPL	Tyrosine-protein phosphatase	45.49
	F9UN21_LACPL	Glycosyltransferase	45.07
	F9UU06_LACPL	Bacteriocin immunity protein PlnI,membrane-bound protease CAAX family	40.98
	F9UN21_LACPL	Glycosyltransferase	37.34
	F9UQ51_LACPL	Polysaccharide biosynthesis polyprenyl glycosylphosphotransferase, priming glycosyltransferase	36.56
	F9UMZ5_LACPL	Polysaccharide biosynthesis protein, chain length regulator	36.23
	F9UMY2_LACPL	Glycosyltransferase (Rhamnosyltransferase),family 2 (GT2)	36.08
	F9UN12_LACPL	Glycosyltransferase, family 2 (GT2)	35.32
	F9UN21_LACPL	Glycosyltransferase	33.86
	F9UU09_LACPL	Bacteriocin ABC-transporter, ATP-binding and permease protein PlnG	33.33
	F9UU09_LACPL	Bacteriocin ABC-transporter, ATP-binding and permease protein PlnG	33.06
	F9UU09_LACPL	Bacteriocin ABC-transporter, ATP-binding and permease protein PlnG	33.02
	F9UN04_LACPL	Glycosyltransferase, family 1 (GT1)	32.67
	F9UU09_LACPL	Bacteriocin ABC-transporter, ATP-binding and permease protein PlnG	32.55
	F9UU09_LACPL	Bacteriocin ABC-transporter, ATP-binding and permease protein PlnG	32.45
	F9UU09_LACPL	Bacteriocin ABC-transporter, ATP-binding and permease protein PlnG	31.11
	F9UU09_LACPL	Bacteriocin ABC-transporter, ATP-binding and permease protein PlnG	30.87
	F9UU09_LACPL	Bacteriocin ABC-transporter, ATP-binding and permease protein PlnG	30.84
	F9UU09_LACPL	Bacteriocin ABC-transporter, ATP-binding and permease protein PlnG	30.73
	F9UN04_LACPL	Glycosyltransferase, family 1 (GT1)	30.43
	F9UU09_LACPL	Bacteriocin ABC-transporter, ATP-binding and permease protein PlnG	30.29
	F9UU09_LACPL	Bacteriocin ABC-transporter, ATP-binding and permease protein PlnG	30.14

	F9UU09_LACPL	Bacteriocin ABC-transporter, ATP-binding and permease protein PlnG	30.09
	F9UU09_LACPL	Bacteriocin ABC-transporter, ATP-binding and permease protein PlnG	30.09
	F9UU09_LACPL	Bacteriocin ABC-transporter, ATP-binding and permease protein PlnG	30.08
L. plantarum SK151	F9UU06_LACPL	Bacteriocin immunity protein PlnI,membrane-bound protease CAAX family	100
	F9UU07_LACPL	Bacteriocin peptide PlnF	100
	F9UU08_LACPL	Bacteriocin peptide PlnE	100
	F9UU09_LACPL	Bacteriocin ABC-transporter, ATP-binding and permease protein PlnG	100
	F9UU10_LACPL	Bacteriocin ABC transporter, accessory factor PlnH	100
	F9UU11_LACPL	Plantaricin biosynthesis protein PlnS	100
	F9UU13_LACPL	Hypothetical membrane protein PlnU, membrane-bound protease CAAX family	100
	F9UU14_LACPL	Hypothetical membrane protein plnV,membrane-bound protease CAAX family	100
	F9UMZ6_LACPL	Polysaccharide biosynthesis protein,regulator	100
	F9UN17_LACPL	Polysaccharide biosynthesis membrane protein	100
	F9UN19_LACPL	Polysaccharide biosynthesis protein	100
	F9UN20_LACPL	O-acetyltransferase	100
	F9USC1_LACPL	Transcription regulator, Cro/CI family	100
	F9UQ50_LACPL	Glycosyltransferase, family 1 (GT1)	99.72
	F9UQ52_LACPL	UDP N-acetyl glucosamine 4-epimerase, NAD dependent	99.68
	F9UMZ5_LACPL	Polysaccharide biosynthesis protein, chain length regulator	99.61
	F9UQ53_LACPL	Tyrosine-protein phosphatase	99.61
	F9UQ51_LACPL	Polysaccharide biosynthesis polyprenyl glycosylphosphotransferase, priming glycosyltransferase	99.55
	F9UQ47_LACPL	Glycosyltransferase, family 2 (GT2)	99.38
	B9V401_LACPN	Bile salt hydrolase	99.38
	F9UN13_LACPL	Glycosyltransferase, family 2 (GT2)	99.03
	F9UQ46_LACPL	Polysaccharide repeat unit transporter (Flippase)	98.96
	F9UN15_LACPL	Polysaccharide biosynthesis protein	98.94
	F9UQ54_LACPL	Polysaccharide biosynthesis protein	98.72
	F9UN16_LACPL	Polysaccharide biosynthesis protein	98.57
	F9UN21_LACPL	Glycosyltransferase	98.45
	F9UQ55_LACPL	Polysaccharide biosynthesis protein, chain length regulator	98.02
	F9UU15_LACPL	Hypothetical membrane protein PlnW,membrane-bound protease CAAX family	97.81
	F9UMZ7_LACPL	Tyrosine-protein phosphatase	97.67

	F9UQ48_LACPL	Polysaccharide polymerase	97.41
	F9UQ49_LACPL	Glycosyltransferase, family 1 (GT1)	96.49
	F9UMZ8_LACPL	UDP N-acetyl glucosamine 4-epimerase, NAD dependent	92.38
	F9UMZ9_LACPL	Priming glycosyltransferase, polyprenyl glycosylphosphotransferase	91.56
	F9UN05_LACPL	Polysaccharide biosynthesis protein	75
	F9UN12_LACPL	Glycosyltransferase, family 2 (GT2)	70.2
	F9UQ52_LACPL	UDP N-acetyl glucosamine 4-epimerase, NAD dependent	60.71
	F9UMX7_LACPL	Oligosaccharide transporter (Flippase)	54.43
	F9USC1_LACPL	Transcription regulator, Cro/CI family	50.88
	F9UU09_LACPL	Bacteriocin ABC-transporter, ATP-binding and permease protein PlnG	48.65
	F9UMZ9_LACPL	Priming glycosyltransferase, polyprenyl glycosylphosphotransferase	41
	F9UN03_LACPL	Oligosaccharide transporter (Flippase)	39.36
	F9USC1_LACPL	Transcription regulator, Cro/CI family	37.88
	F9USC1_LACPL	Transcription regulator, Cro/CI family	35.71
	F9UU09_LACPL	Bacteriocin ABC-transporter, ATP-binding and permease protein PlnG	35.68
	F9UU06_LACPL	Bacteriocin immunity protein PlnI,membrane-bound protease CAAX family	35.42
	F9USC1_LACPL	Transcription regulator, Cro/CI family	35.38
	F9UU09_LACPL	Bacteriocin ABC-transporter, ATP-binding and permease protein PlnG	34.94
	F9UU09_LACPL	Bacteriocin ABC-transporter, ATP-binding and permease protein PlnG	34.27
	F9UU06_LACPL	Bacteriocin immunity protein PlnI,membrane-bound protease CAAX family	33.93
	F9UN03_LACPL	Oligosaccharide transporter (Flippase)	33.86
	F9UU09_LACPL	Bacteriocin ABC-transporter, ATP-binding and permease protein PlnG	33.77
	F9USC1_LACPL	Transcription regulator, Cro/CI family	33.33
	F9UU15_LACPL	Hypothetical membrane protein PlnW,membrane-bound protease CAAX family	32.48
	B9V401_LACPN	Bile salt hydrolase	32.44
	F9UU09_LACPL	Bacteriocin ABC-transporter, ATP-binding and permease protein PlnG	32.31
	F9UQ47_LACPL	Glycosyltransferase, family 2 (GT2)	31.98
	F9UU09_LACPL	Bacteriocin ABC-transporter, ATP-binding and permease protein PlnG	31.96
	F9UU09_LACPL	Bacteriocin ABC-transporter, ATP-binding and permease protein PlnG	31.8
	F9UU09_LACPL	Bacteriocin ABC-transporter, ATP-binding and permease protein PlnG	31.58
	F9UU09_LACPL	Bacteriocin ABC-transporter, ATP-binding and permease protein PlnG	31.58
	F9UU09_LACPL	Bacteriocin ABC-transporter, ATP-binding and permease protein PlnG	31.48

	F9USC1_LACPL	Transcription regulator, Cro/CI family	31.34
	F9UU09_LACPL	Bacteriocin ABC-transporter, ATP-binding and permease protein PlnG	31.1
	F9UU09_LACPL	Bacteriocin ABC-transporter, ATP-binding and permease protein PlnG	30.97
	F9UU09_LACPL	Bacteriocin ABC-transporter, ATP-binding and permease protein PlnG	30.7
	F9UU09_LACPL	Bacteriocin ABC-transporter, ATP-binding and permease protein PlnG	30.7
	F9UU09_LACPL	Bacteriocin ABC-transporter, ATP-binding and permease protein PlnG	30.58
	F9UU09_LACPL	Bacteriocin ABC-transporter, ATP-binding and permease protein PlnG	30.37
	F9UU09_LACPL	Bacteriocin ABC-transporter, ATP-binding and permease protein PlnG	30.37
	F9UU09_LACPL	Bacteriocin ABC-transporter, ATP-binding and permease protein PlnG	30.33
	Q88ZC1_LACPL	Hypothetical membrane protein PlnT,membrane-bound protease CAAX family	30.21
	B9V401_LACPN	Bile salt hydrolase	30.21
	F9UMZ8_LACPL	UDP N-acetyl glucosamine 4-epimerase, NAD dependent	30.17
	F9UU09_LACPL	Bacteriocin ABC-transporter, ATP-binding and permease protein PlnG	30
L. fermentum SK152	F9UMZ9_LACPL	Priming glycosyltransferase, polyprenyl glycosylphosphotransferase	75.45
	F9UMX8_LACPL	Glycosyltransferase	57.6
	F9UQ52_LACPL	UDP N-acetyl glucosamine 4-epimerase, NAD dependent	57.52
	F9UMZ6_LACPL	Polysaccharide biosynthesis protein,regulator	51.92
	F9UMX7_LACPL	Oligosaccharide transporter (Flippase)	45.44
	F9UMZ5_LACPL	Polysaccharide biosynthesis protein, chain length regulator	44.09
	F9UMZ7_LACPL	Tyrosine-protein phosphatase	43.75
	F9UMZ9_LACPL	Priming glycosyltransferase, polyprenyl glycosylphosphotransferase	42.71
	F9UMZ9_LACPL	Priming glycosyltransferase, polyprenyl glycosylphosphotransferase	40.91
	CPS2G_LACPL	Exopolysaccharide phosphotransferase cps2G	40.12
	F9UU09_LACPL	Bacteriocin ABC-transporter, ATP-binding and permease protein PlnG	35.34
	F9USC1_LACPL	Transcription regulator, Cro/CI family	34.74
	F9UU13_LACPL	Hypothetical membrane protein PlnU,membrane-bound protease CAAX family	34.26
	F9UU09_LACPL	Bacteriocin ABC-transporter, ATP-binding and permease protein PlnG	33.93

	F9UU09_LACPL	Bacteriocin ABC-transporter, ATP-binding and permease protein PlnG	33.05
	F9UN13_LACPL	Glycosyltransferase, family 2 (GT2)	32.87
	F9UU09_LACPL	Bacteriocin ABC-transporter, ATP-binding and permease protein PlnG	32.22
	F9USC1_LACPL	Transcription regulator, Cro/CI family	32.1
	F9UQ47_LACPL	Glycosyltransferase, family 2 (GT2)	31.73
	F9UU06_LACPL	Bacteriocin immunity protein PlnI,membrane-bound protease CAAX family	31.25
	F9UU09_LACPL	Bacteriocin ABC-transporter, ATP-binding and permease protein PlnG	31.21
	F9UU09_LACPL	Bacteriocin ABC-transporter, ATP-binding and permease protein PlnG	31.08
	F9UU09_LACPL	Bacteriocin ABC-transporter, ATP-binding and permease protein PlnG	30.88
	F9USC1_LACPL	Transcription regulator, Cro/CI family	30.77
	F9UU09_LACPL	Bacteriocin ABC-transporter, ATP-binding and permease protein PlnG	30.66
	F9UU09_LACPL	Bacteriocin ABC-transporter, ATP-binding and permease protein PlnG	30.57
	F9UU09_LACPL	Bacteriocin ABC-transporter, ATP-binding and permease protein PlnG	30.56
	F9UU09_LACPL	Bacteriocin ABC-transporter, ATP-binding and permease protein PlnG	30.26
	F9USC1_LACPL	Transcription regulator, Cro/CI family	30.16
L. johnsonii PF01	F9UU09_LACPL	Bacteriocin ABC-transporter, ATP-binding and permease protein PlnG	62.34
	B9V401_LACPN	Bile salt hydrolase	53.16
	B9V401_LACPN	Bile salt hydrolase	49.84
	F9UMZ7_LACPL	Tyrosine-protein phosphatase	47.06
	F9UMX7_LACPL	Oligosaccharide transporter (Flippase)	47.03
	F9UMZ6_LACPL	Polysaccharide biosynthesis protein,regulator	45.58
	F9UU09_LACPL	Bacteriocin ABC-transporter, ATP-binding and permease protein PlnG	43.94
	F9UMZ9_LACPL	Priming glycosyltransferase, polyprenyl glycosylphosphotransferase	42.34
	F9UU10_LACPL	Bacteriocin ABC transporter, accessory factor PlnH	38.78
	B9V401_LACPN	Bile salt hydrolase	38.36
	F9USC1_LACPL	Transcription regulator, Cro/CI family	37.5
	F9UMX6_LACPL	Glycosyltransferase, family 1 (GT1)	35.82
	F9UU09_LACPL	Bacteriocin ABC-transporter, ATP-binding and permease protein PlnG	35.59
	F9UU10_LACPL	Bacteriocin ABC transporter, accessory factor PlnH	34.26
	F9UU09_LACPL	Bacteriocin ABC-transporter, ATP-binding and permease protein PlnG	33.91
	Q88ZC1_LACPL	Hypothetical membrane protein PlnT,membrane-bound protease CAAX family	33.33

	F9UU09_LACPL	Bacteriocin ABC-transporter, ATP-binding and permease protein PlnG	33.05
	F9UU09_LACPL	Bacteriocin ABC-transporter, ATP-binding and permease protein PlnG	33.05
	F9UU09_LACPL	Bacteriocin ABC-transporter, ATP-binding and permease protein PlnG	32.91
	F9UU09_LACPL	Bacteriocin ABC-transporter, ATP-binding and permease protein PlnG	32.73
	F9UQ50_LACPL	Glycosyltransferase, family 1 (GT1)	32.62
	F9UU09_LACPL	Bacteriocin ABC-transporter, ATP-binding and permease protein PlnG	32.42
	F9UU09_LACPL	Bacteriocin ABC-transporter, ATP-binding and permease protein PlnG	32.2
	F9UMY2_LACPL	Glycosyltransferase (Rhamnosyltransferase),family 2 (GT2)	32
	F9UU09_LACPL	Bacteriocin ABC-transporter, ATP-binding and permease protein PlnG	31.88
	F9UMZ8_LACPL	UDP N-acetyl glucosamine 4-epimerase, NAD dependent	31.85
	F9UU09_LACPL	Bacteriocin ABC-transporter, ATP-binding and permease protein PlnG	31.74
	F9UU09_LACPL	Bacteriocin ABC-transporter, ATP-binding and permease protein PlnG	31.67
	F9UU09_LACPL	Bacteriocin ABC-transporter, ATP-binding and permease protein PlnG	31.58
	F9UMY2_LACPL	Glycosyltransferase (Rhamnosyltransferase),family 2 (GT2)	31.45
	F9UU09_LACPL	Bacteriocin ABC-transporter, ATP-binding and permease protein PlnG	31.44
	F9UU09_LACPL	Bacteriocin ABC-transporter, ATP-binding and permease protein PlnG	31.36
	F9UU09_LACPL	Bacteriocin ABC-transporter, ATP-binding and permease protein PlnG	30.93
	F9UQ55_LACPL	Polysaccharide biosynthesis protein, chain length regulator	30.89
	F9UU09_LACPL	Bacteriocin ABC-transporter, ATP-binding and permease protein PlnG	30.73
	F9UU09_LACPL	Bacteriocin ABC-transporter, ATP-binding and permease protein PlnG	30.65
	F9UU09_LACPL	Bacteriocin ABC-transporter, ATP-binding and permease protein PlnG	30.46
	F9UU09_LACPL	Bacteriocin ABC-transporter, ATP-binding and permease protein PlnG	30.37
	F9UU09_LACPL	Bacteriocin ABC-transporter, ATP-binding and permease protein PlnG	30.06
	F9UQ47_LACPL	Glycosyltransferase, family 2 (GT2)	30
	F9USC1_LACPL	Transcription regulator, Cro/CI family	30
